# P01.22. Identification of a peptide biomarker from bromelain, an extract of ananas comosus merr, using LC-SRM/MS

**DOI:** 10.1186/1472-6882-12-S1-P22

**Published:** 2012-06-12

**Authors:** E Secor, S Szczepanek, L Guernsey, L Silbart, R Thrall, D Han

**Affiliations:** 1 University of Connecticut Health Center, Farmington, USA; 2 Neag Cancer Center, UCHC, Farmington, USA; 3 Department of Allied Health Sciences, Storrs, USA; 4 Center for Vascular Biology, UCHC, Farmington, USA

## Purpose

Bromelain, a pineapple extract, is a complex mixture of proteases and protease inhibitors. Although bromelain (Br) has been used clinically for over 40 years, the active constituents that mediate its anti-inflammatory activity are not thoroughly characterized and no biomarker exists to evaluate absorbed peptides and their potential therapeutic responses.

## Methods

Stem Br (Vital Nutrients lot # 2890; Middletown, CT), was tested for authenticity and purity. Female C57BL/6J mice, (Jackson Laboratory; Bar Harbor, ME) received one bolus, i.p. injection of Br (12mg/kg) in 0.5ml of physiological saline and plasma collected at baseline 3, 6, 12 and 24 hours. All procedures were approved by the Animal Care Committee at University of CT Health Center. Post SDS-PAGE in gel digestion, samples (Br raw material, spiked plasma and plasma from i.p. teated animals) were analyzed via LC/MS/MS (Liquid Chromatography-Mass Spectrometry-Mass) and LC-SRM/MS (Liquid Chormatography-Selected Reaction Monitoring/Mass Spectrometry). Data were searched using Mascot and Scaffold algorithms.

## Results

Within the Br raw material we identified 44 proteins of which several were Br-specific including nine proteases, one glycosidase and three protease inhibitors. In Br spiked plasma, 7 Br-specific proteins (ananain, Br inhibitor, comosain, cysteine proteinase precursor ANll, FB1035 precursor, FBSB precursor and jacalin-like lectin), similar to those found in the Br raw material, were identified. Within these 7 proteins, 21 Br-specific peptides were further identified and characterized based on their ion spectrum and fragmentation patterns including the unique peptide, DYGAVNEVK. Using SRM DYGAVNEVK was identified in plasma of Br-treated mice. The spectral count of DYGAVNEVK peaked at 6 hours and was undetectable by 24 hours.

## Conclusion

This Br peptide could serve as a biomarker to standardize the therapeutic dose or Br and maximize its clinical utility.

